# Isolation and characterizations of a novel recombinant scFv antibody against exotoxin A of *Pseudomonas aeruginosa*

**DOI:** 10.1186/s12879-021-05969-0

**Published:** 2021-03-24

**Authors:** Zahra Shadman, Safar Farajnia, Mohammad Pazhang, Mohammadreza Tohidkia, Leila Rahbarnia, Saeed Najavand, Sayna Toraby

**Affiliations:** 1 Drug Applied Research Center, Tabriz University of Medical Sciences, Tabriz, Iran; 2 Department of Cellular and Molecular Biology, Faculty of Science Faculty, Azarbaijan Shahid Madani University, Tabriz, Iran; 3 Biotechnology Research Center, Tabriz University of Medical Sciences, Tabriz, Iran; 4 Nanotechnology Research Center, Tabriz University of Medical Sciences, Tabriz, Iran; 5 Infectious and Tropical Diseases Research Center, Tabriz University of Medical Sciences, Tabriz, Iran

**Keywords:** Phage display, *Pseudomonas aeruginosa*, Exotoxin A, Human single chain antibody

## Abstract

**Background:**

*Pseudomonas aeruginosa* is the leading cause of nosocomial infections, especially in people with a compromised immune system. Targeting virulence factors by neutralizing antibodies is a novel paradigm for the treatment of antibiotic-resistant pseudomonas infections. In this respect, exotoxin A is one of the most potent virulence factors in *P. aeruginosa.* The present study was carried out to identify a novel human scFv antibody against the *P. aeruginosa* exotoxin A domain I (ExoA-DI) from a human scFv phage library.

**Methods:**

The recombinant ExoA-DI of *P. aeruginosa* was expressed in *E. coli*, purified by Ni-NTA column, and used for screening of human antibody phage library. A novel screening procedure was conducted to prevent the elimination of rare specific clones. The phage clone with high reactivity was evaluated by ELISA and western blot.

**Results:**

Based on the results of polyclonal phage ELISA, the fifth round of biopanning leads to the isolation of several ExoA-DI reactive clones. One positive clone with high affinity was selected by monoclonal phage ELISA and used for antibody expression. The purified scFv showed high reactivity with the recombinant domain I and full-length native exotoxin A.

**Conclusions:**

The purified anti-exotoxin A scFv displayed high specificity against exotoxin A. The human scFv identified in this study could be the groundwork for developing a novel therapeutic agent to control *P. aeruginosa* infections.

## Background

*Pseudomonas (p.) aeruginosa* is the most common cause of nosocomial infections leading to a high mortality rate, especially in people with cystic fibrosis, neoplastic disease, and severe burns [1]. Currently, the outbreak of antibiotic resistant strains has become one of the serious challenges for global health. Toxins play an essential role in bacterial pathogenesis. *P. aeruginosa* produces several types of toxins, including exotoxin A, phosphorylase C, hemolysin, and exoenzyme S which among them, exotoxin A plays a major role in the progress and prognosis of *P. aeruginosa* infections. Exotoxin A is a single-chain polypeptide with a molecular weight of 66.583, consisting of 613 amino acids comprised of three domains [2, 3]. Domain I is responsible for the attachment of toxin to the cell receptor and comprising two subdomain Ia (amino acids 1 to 252) and Ib (amino acids 365 to 404). The function of domain Ib is not well characterized, but it may be necessary for the secretion or activity of the toxin. The second domain comprises amino acids 253 to 364, consists of 6 alpha helix sequences, and is essential for transferring the toxin over the membrane. The third domain (405 to 613 amino acids) is the enzymatic and catalytic domain with ADP ribosyl transfer activity, which inhibits protein synthesis ultimately results in cell death [4]. In addition to the above mentioned domains, there are two crucial motifs inside exotoxin A. The first motif (280–274 RHRQPRG amino acids) is located in domain II, appears on the toxin’s external surface, and is broken down by the eukaryotic proteases. The second motif (the REDLK-591913–609) is located at the toxin’s carboxylic end and is responsible for retaining the toxin in the endoplasmic reticulum compartment. Both motifs are essential for toxicity [4]. It has shown that antibodies against Exotoxin A can significantly increase the survival rate of infected subjects [5]. Hence the development of anti-exotoxin A antibody is of great interest for the treatment of pseudomonas infections.

At present, human scFv phage libraries have provided a quick and reliable approach to develop human antibodies against almost any antigen. This study aimed to identify a human anti-exotoxin A scFv and evaluate its specificity to *P. aeruginous* exotoxin A.

## Methods

### scFv-phage library, bacterial strains, and components

The semisynthetic human scFv phage libraries I & J (Tomlinson I J), *E. coli* strains (HB2151 and TG1), and *KM13* helper phage were from the Medical Research Council (MRC), Cambridge. *E. coli* BL21(DE3) were from Novagene [6, 7].

### Expression and purification of exotoxin A domain I

*E. coli* containing the ExoA-DI encoding construct were cultured in LB media and used for plasmid extraction by FAVORGEN plasmid extraction kit according to the manufacturer instruction. The expression construct was then transformed into *E. coli* BL21, cultured in LB media and induced with IPTG (0.5 mM). For optimized expression, we examined different induction times, and 21 h was selected as the best induction time. The expression was assessed by SDS-PAGE. For purification, the bacteria were cultured in 200 ml volume at 37 °C and lysed by sonication. The inclusion body was washed by washing buffer, solubilized in 8 M urea, and purified by Ni-NTA column. After that, the recombinant ExoA-DI was refolded by stepwise removal of urea by dialysis [8].

### scFv phage library screening

After the amplification of Tomlinson I library, it was screened for six rounds against ExoA-DI protein [9]. The biopanning process was started with 100 μg/ml ExoA-DI on a Maxisorb 96-well plate in PBS buffer [10]. During biopanning rounds, to prevent the elimination of specific rare clones, the concentration of domain I protein was kept constant [10–12].

To increase the screening stringency, the time of incubation of phage pool with antigen was decreased and washing numbers between screening rounds were increased (Table 1). In the beginning, the domain I - immobilized plate was blocked with 3% Bovine Serum Albumin (BSA) for 2 h, then 10 ^12–13^ pfu phages were added into the plates. After the incubation for 60 min at room temperature (RT), the plate was washed with PBS containing 0.1% Tween 20 (PBS-T), and trypsin-PBS (100 μl of 10 mg/ml trypsin stock solution in10 ml PBS) was used for elution of the bound phages (Table 1). The biopanning rounds were continued to reach the maximum OD in polyclonal phage ELISA. Totally, six rounds of biopanning were carried out to select domain I - specific phage clones. However, the biopanning was done until the sixth round; the results increased until the fifth round of enrichment. In the sixth round, the rate of enrichment was decreased, which indicated the completion of biopanning.

**Table 1:** Data related to antigen concentrations, blocking buffers, Tween 20 percentage and washing numbers during six biopanning rounds

Rounds	I	II	III	IV	V	VI	VII
Protein (μg/ml)	375	250	150	120	150	150	150
Blocking buffers	%2 skim milk	%3 BSA	%2 skim milk	%3 BSA	%2 skim milk	%3 BSA	%2 skim milk
% Tween 20	%0.1	%0.1	%0.1	%0.1	%0.1	%0.1	%0.1
Washing numbers	3	20	10	20	20	20	25
Phage incubation time (hrs)	2	2	2	2	2	2	2

### Investigation of the specificity of the phage clones by polyclonal phage ELISA

To investigate the specificity of selected phages from each round, a polyclonal phage ELISA against ExoA-DI was performed. For this purpose, 60 μg/ml ExoA-DI was coated into the ELISA plates and incubated at 4 °C overnight. After blocking with 3% BSA for 1 h, the eluted phages from each round (1:10 dilutions in 1% BSA-PBS) were added to the plates and incubated for 1 h RT. After that, the plates were incubated with anti-M13- HRP (1:2000 dilutions in 1% BSA-PBS) for 1 h. The reactivity was determined using TMB substrate. The optical density was recorded via ELISA Reader at 450 nm.

### Selection of scFvs clones to ExoA-DI

The single colonies were randomly selected from the fifth round of screening and used to identify specific scFv clones by monoclonal phage ELISA. For this purpose, the individual colonies were inoculated into 2xTY medium (1% [w/v] yeast extract, 1.6% [w/v] tryptone, and 0.5% [w/v] sodium chloride) containing 4% glucose and 100 μg/ml ampicillin in a 96 well plate. The plate was incubated at 37 °C for 2 h [18, 19]. After adding 10^9^ helper phages to the wells and incubating for 1 h at 37 °C, the plate was centrifuged at 3000 xg for 10 min. The supernatants were aspirated off and the bacterial pellet was resuspended in 2xTY medium containing 50 μg/ml kanamycin and 100μg/ml ampicillin. The cultures were continued overnight at 30 °C in a shaking incubator (250 rpm) and culture supernatants (1:2 dilution in1% BSA-PBS) were utilized for phage ELISA as described above.

### Screening of clones via soluble fragment ELISA

All positive clones were initially confirmed by PCR with LMB3 and PHEN specific primers shown in Table 2 [13]. An scFv ELISA was used to evaluate the specificity of positive phage clones against Exo A DI. For this, a positive phage clone with high OD in phage ELISA was cultured in 2xTY medium containing ampicillin and 0.1% glucose at 37 °C. The expression of antibody was induced by 0.5 mM IPTG at OD600 = 0.9, and the culture was continued at 200 rpm overnight at 37 °C. The soluble scFvs were collected from the periplasmic fraction and used for ELISA. For this purpose, the ELISA plate was coated with ExoA-DI (60 μg/ml overnight at 4 °C), and blocked with 3% BSA for 2 h and washed with PBS-T. Then soluble scFvs were added to the plate in different dilutions, incubated for 1 h, and washed with PBS-T. The plate was incubated with HRP conjugated Protein L (1:2000 dilutions in PBS-% BSA) for 1 h. The reaction was finally developed by TMB substrate, and the OD values were measured with ELISA reader at 450 nm.

**Table 2:** The sequence of primers used for sequencing of scFv clones

Primer		Sequence
Phen	Reverse	5′-CAG GAA ACA GCT ATG AC-3’
LMB3	Forward	5′-CTA TGC GGC CCC ATT CA-3’

### Expression of soluble anti exotoxin A scFv in *E. coli*

To increase the expression of selected scFv, the sequence coding for scFv fragment was amplified by PCR and subcloned into the *pET28a* expression vector. The construct was transformed into *E. coli* BL21 (DE3) pLysS strain and cultured in LB media containing kanamycin (5mcg/ml). The antibody expression was induced by 0.5 mM IPTG at OD600 = 0.7, culture was continued overnight at 22 °C in a shaking incubator, and the expression was assessed by SDS-PAGE. The supernatant containing secreted antibody fragments was purified by Ni-NTA column and confirmed by 12% SDS-PAGE. Then the reactivity of the purified scFv was checked by the ELISA technique.

### Assessment of the reactivity of recombinant scFv with exotoxin A

The specificity of the purified scFv to ExoA-DI was evaluated by western blot. For this, 2.5 μg of purified ExoA-DI and 1 μg of native exotoxin A were electrophoresed through a 12% SDS-PAGE and transferred onto PVDF membrane (Invitrogen, USA). The membrane was blocked with 5% Skim Milk-PBS overnight at 4 °C and incubated with the purified scFv followed by adding HRP conjugated Protein L (1–1000 dilutions) as the secondary antibody. The reaction was developed by using the DAB substrate.

### Production of *P. aeruginosa* native exotoxin A

For the production of native exotoxin A, the trypticase soy broth culture medium was dialyzed against 0.01 M Tris buffer, PH 8 at 4 °C for 24 h. Then, the dialysate was sterilized by autoclave and enriched by 1% glycerol and 1 M monosodium glutamate. *P. aeruginosa* strain PAO1 was cultured in this media at 32 °C for 22 h. Finally, the culture supernatant was concentrated with ammonium sulfate [14–16].

### Evaluation of the reactivity of recombinant scFV antibody with native exotoxin A

The purified exotoxin A was coated in an ELISA plate overnight. Then, different dilutions of recombinant scFv antibody were added into the wells, and the reaction was determined according to the ELISA method described earlier.

## Results

### Enrichment and specificity of anti-exotoxin A phages during biopanning rounds

The enrichment rate of specific phages against ExoA-DI was calculated during *five rounds of biopanning*. As expected, in the first round of screening, the yield of specific phages to ExoA-DI was very low while, from the 3rd round, the elimination of nonspecific phages led to the enrichment of specific phages to 0.72*10^− 4^ that continued during rounds 4 and 5 (Table 3). Totally, the eluted phages were increased from 1.40*10^6^ in the second round to 7.27*10^8^ in the fifth round. However, the enrichment rate was again reduced in the 6th round. These findings were indicating an effective screening to obtain specific phages against ExoA-DI. A polyclonal phage ELISA was performed after each biopanning round to examine the specificity of the eluted phages against ExoA-DI.

**Table 3:** The input, output and input/output ratio of scFv phage clones during different rounds of biopanning

Round	Input (pfu)	Output (pfu)	Enrichment (output/input)
**1**	3.52*10^12^	1.40*10^6^	0.39*10^−6^
**2**	1.40*10^12^	1.94*10^9^	1.38*10^−3^
**3**	2.52*10^12^	1.83*10^8^	0.72*10^−4^
**4**	1.62*10^12^	1.80*10^8^	1.11*10^−4^
**5**	2.908*10^13^	7.27*10^8^	2.5*10^−5^

**Fig. 1:**
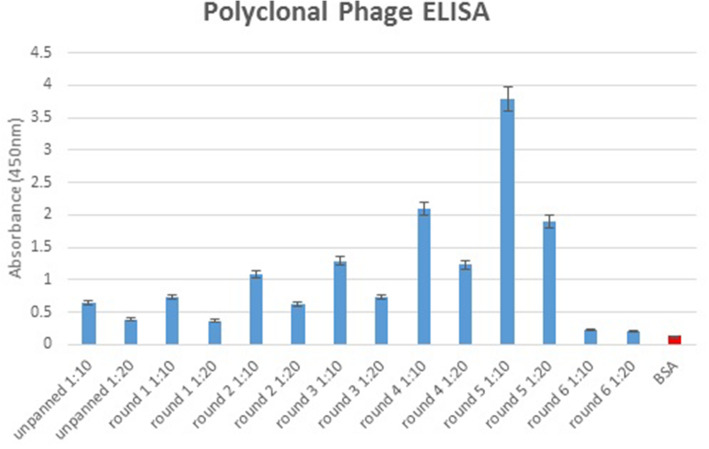
Results of polyclonal phage ELISA related to six biopanning rounds against anti ExoA- DI protein. The plates were coated with 24 μg/ml domain I protein, blocked with 3% BSA, and the binding of precipitated phages from different rounds was determined using anti-M13-HRP at 1:2000 dilution. Absorbance values are represented as the mean ± standard deviation (SD) of three independent determinations. Error bars show the standard deviation for each set of data

The BSA was used as the negative control and displayed a weak signal in ELISA (Fig. 1). Based on the results, the lowest OD was related to the wild-type phages before panning. After six rounds of biopanning, the 5th round indicated the highest binding reactivity to ExoA-DI, so it was selected for further studies.

### Determination of the binding of scFv phage clones to ExoA-DI by monoclonal phage ELISA

**Fig. 2:**
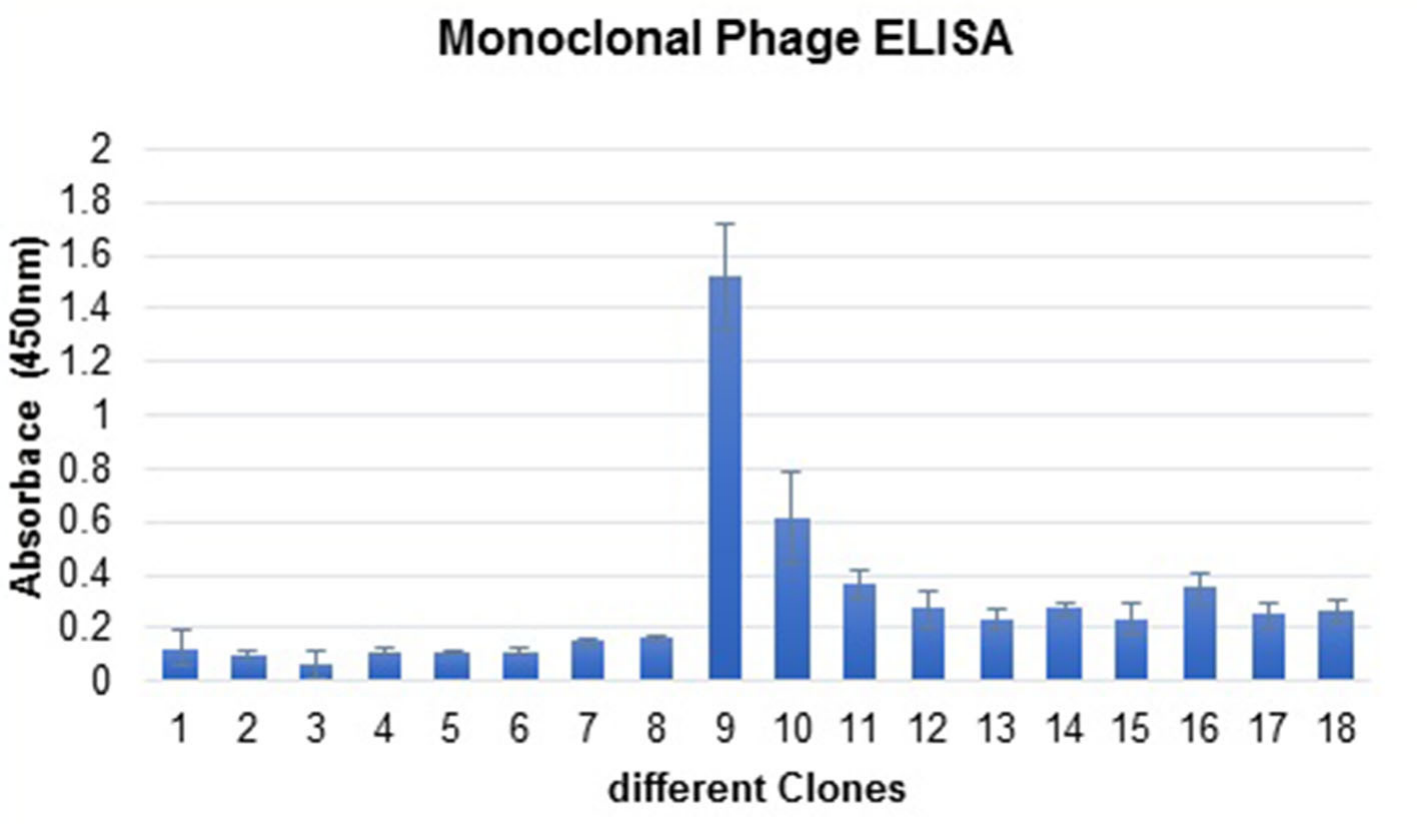
The specificity of the selected phage clones for ExoA-DI. To perform a monoclonal phage ELISA, 60 μg/ml of ExoA-DI was coated per well of ELISA plates, and 100 μl supernatant of each phage clone was separately added per well. Clone number 1 related to the wild-type phage that was used as the negative control. The reactivity was assessed using anti-M13-HRP at 1:2000 dilutions. Absorbance values were recorded as the mean ± SD for three independent determinations. Error bars are indicating the standard deviation for each set of data

**Fig. 3:**
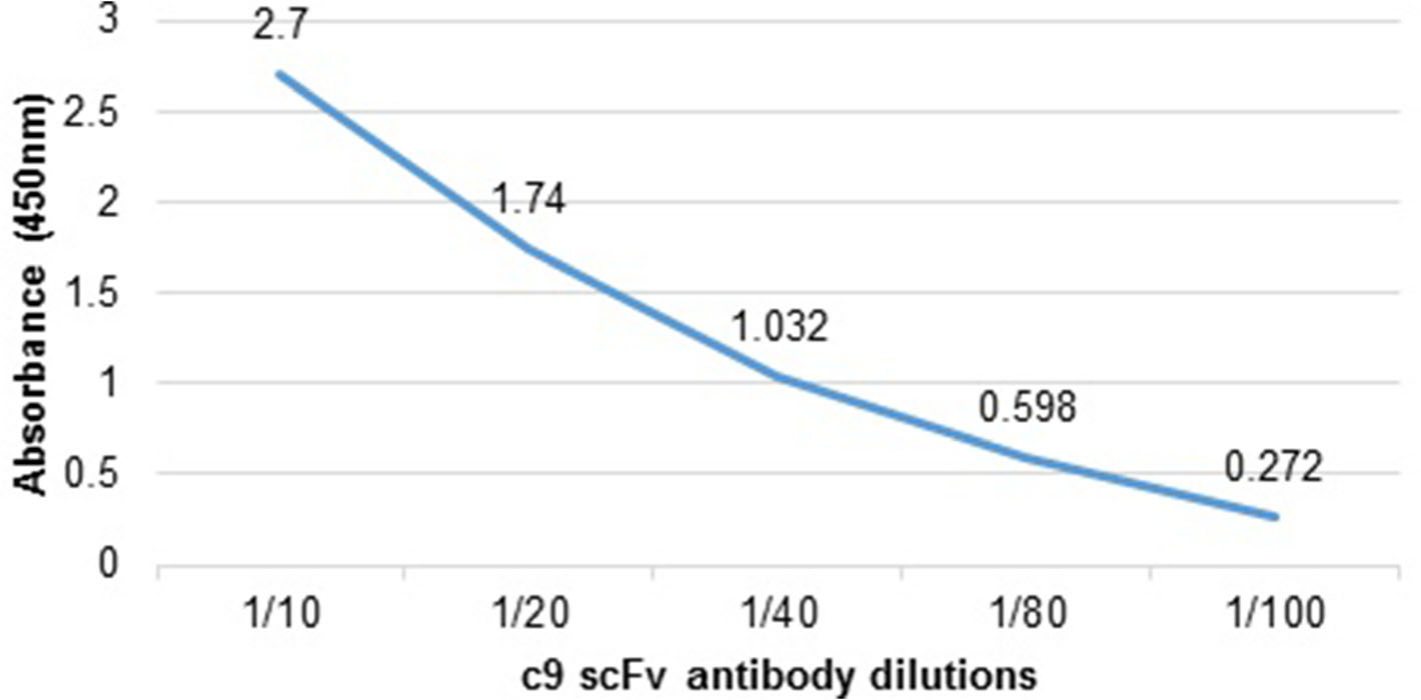
Comparison of the reactivity of different dilution of C9 scFv phage clone with ExoA-DI

We examined more than 100 phage clones randomly from the fifth round, among which 18 specific phage clones were identified by monoclonal phage ELISA with optical densities 0.12 to 1.533 showing significant differences in the binding activity of selected phage clones. Among positive clones, one of the positive phage clones (C9) with higher reactivity to the domain I was used for further analysis (Fig. 2). The reactivity of serialy diluted C9 scFv against ExoA-DI revealed the high affinity of selected antibody clone (Fig. 3).

### Sequence analysis of the positive clones

**Fig. 4:**
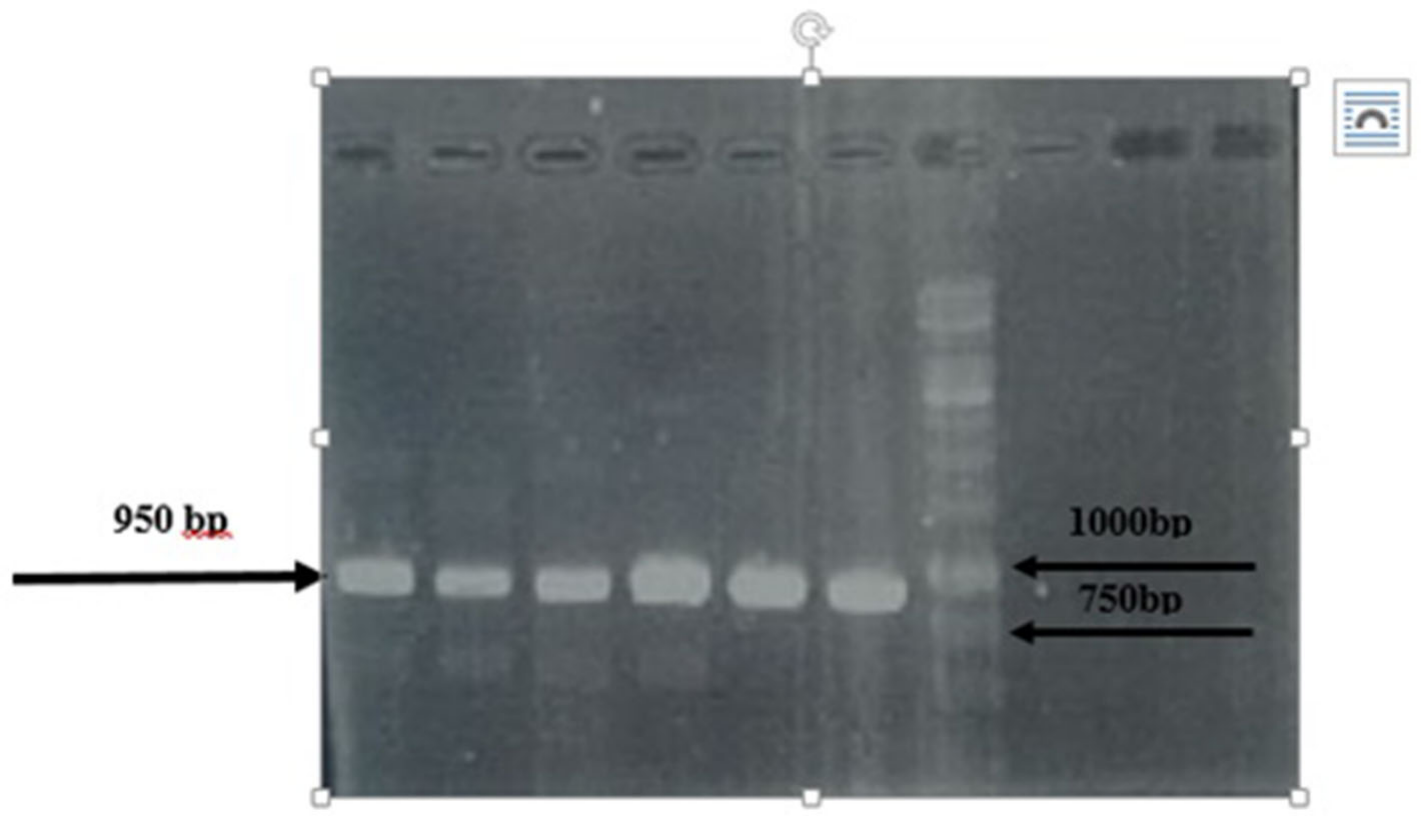
PCR analysis of positive phage clones using vector-specific primers along with DNA size marker (1 kb size marker, Fermentas SM031)

To confirm the presence of both VH and VL fragments in the positive clones, DNA was extracted from the 18 positive clones. PCR screening using PHEN and LMB3 specific primers results in amplifying a specific PCR fragment that appeared as a single 950 bp band (Fig. 4).

**Fig. 5:**
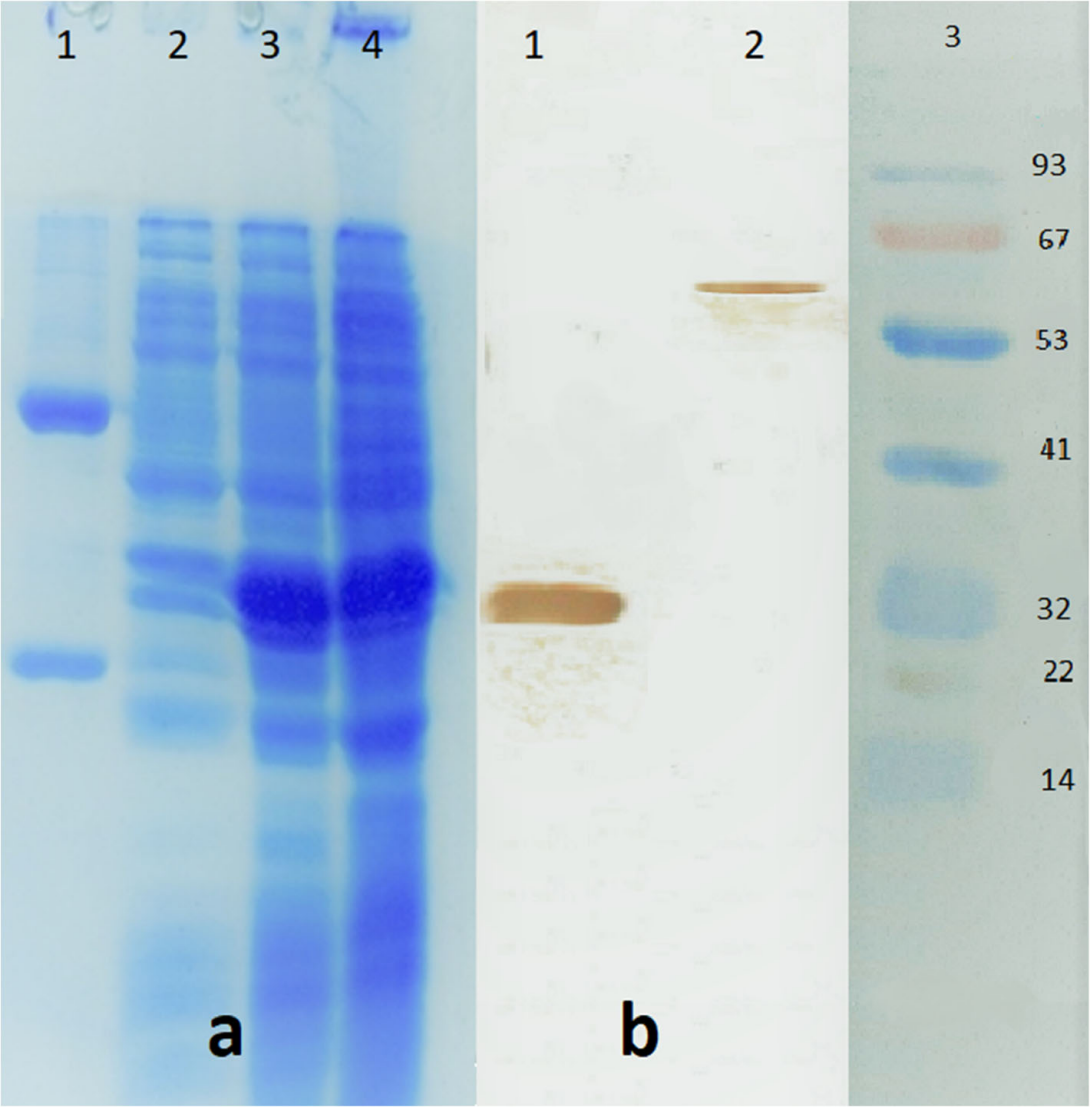
**a** SDS-PAGE analysis of anti ExoA-DI expression in *E. coli*. The results revealed a high level expression of scFv. HB2151 containing scFv inserts were grown and induced with IPTG for scFv secretion. Lane1, IgG; Lane 2, c9 scFv clone before induction; lane 3, 16 h hour after induction, lane 4, 22 h after induction. **b** Western blot analysis of the selected scFv antibody against ExoA-DI and native exotoxin A. Exo-DI and native exotoxin A was electrophoresed through a 12% SDS-polyacrylamide gel and transferred onto PVDF membrane along with a pre-stained protein size marker (abcam cat no. ab116027). The membrane was incubated with HRP conjugated protein L and developed using DAB substrate. The size of scFv was observed in the expected size of about 30 KD and 63 kDa. Lane 1, purified Exo A DI; Lane 2, Native exotoxin A; Lane 3, pre-stained size marker

In the next step, the integrity of the C9 scFv clone with the highest affinity was confirmed by sequencing. Blast analysis indicated that the nucleotide sequence is related to human VH and VL fragments. There was no amber stop codon and mutation in the selected clone. Analysis of CDR regions was performed by http://www.imgt.org/3Dstructure-DB/cgi/DomainGapAlign.cgi and displayed in Table 4.

**Table 4:** Sequence analysis of the C9 scFv phage clone and its CDRs region

C9 sequence	MKYLLPTAAAGLLLLAAQPAMAEVQLLESGGGLVQPGGSLRLSCAASGFTFSSYAMSWVRRAPGKGLEWVSTISSSGSATSYADSVKGRFTISRDNSKNTLYLQMNSLRAEDTAVYYCAKTASSFDYWGQGTLVTVSSGGGGSGGGGSGGGGSTDIQITQSPSSLSASVGDRVTITCRASQSISSYLNWYQQKPGKAPKLLIYNASYLQSGVPSRFSGSGSGTDFTLTISSLQPEDFATYYCQQSNAGPTTFGQGTKVEIKRAAAHHHHHHGAAEQKLISEEDLNGAA	
VL/VH	VL	VH
**CDR1**	GFTFSSYA	QSISSY
**CDR2**	ISSSGSAT	NASYLQSGVPS
**CDR3**	AKTASSFDY	QQSNAGPTT

### Assessment of the reactivity of selected scFv by western blotting

After purification of the selected scFv (c9) by Ni-NTA column, its purity was confirmed by SDS-PAGE, which showed a single band of 25 kDa. The reactivity of scFv to ExoA-DI was assessed by western blot. Results indicated a single band with a molecular weight (MW) of about 30 kDa (Fig. 5), which was consistent with the calculated MW for the ExoA-DI protein [17].

### Reactivity of recombinant scFV antibody with native exotoxin A

**Fig. 6:**
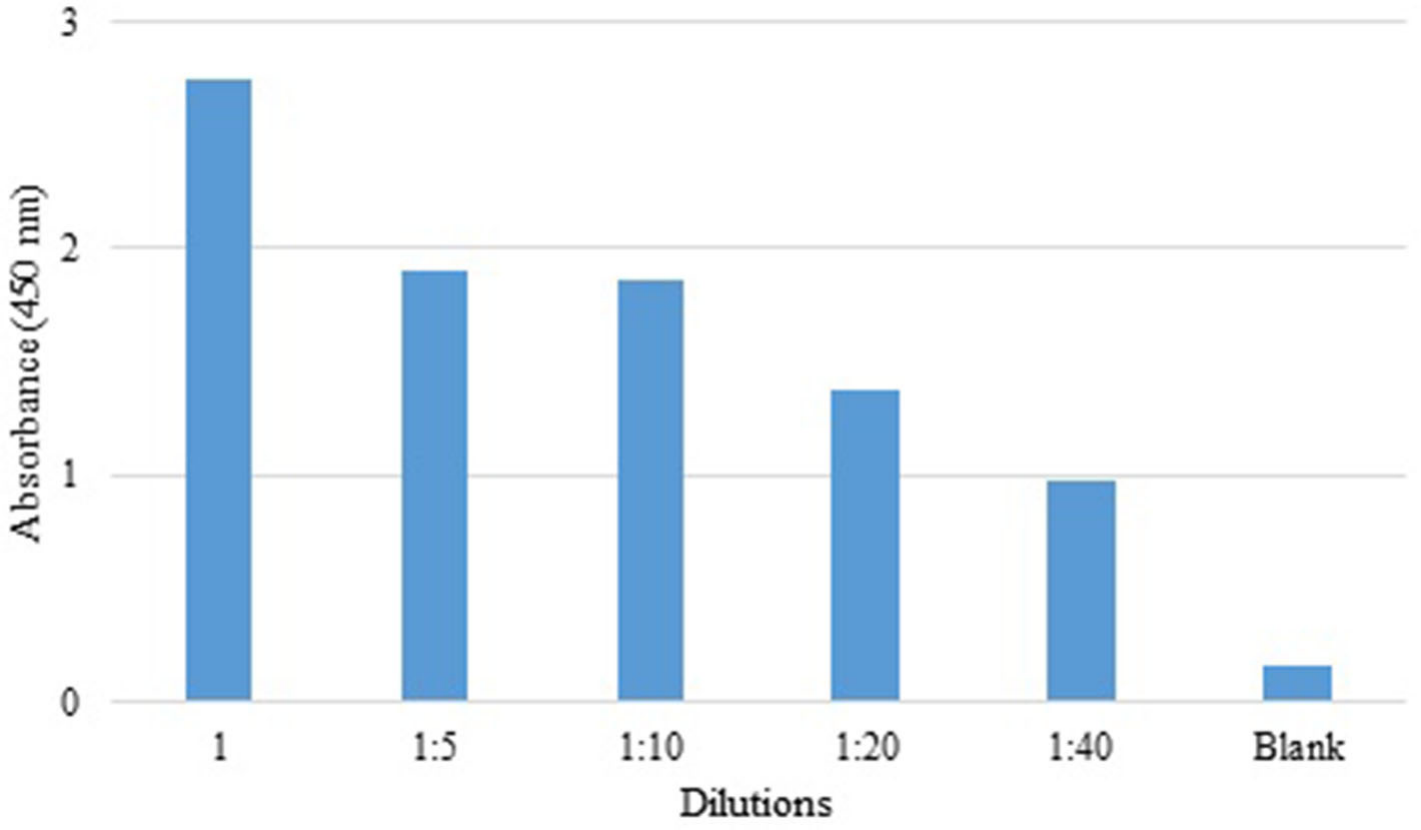
The reactivity of different dilutions of C9 scFv antibody against native exotoxin A by ELISA method. The results indicated high reactivity of selected scFv antibody with native exotoxin A

Analysis of the reactivity of clone 9 scFv antibody with purified native exotoxin A by ELISA showed that the scFv antibody could detect native exotoxin with high affinity (Fig. 6). This result indicates that the antibody produced against the domain I also had a significant reactivity with the native toxin.

## Discussion

Exotoxin A is one of the most potent virulence factors in *P. aeruginosa*. With the increasing prevalence of multi drug resistant strains, it is necessary to develop alternative therapeutic approaches to combat resistant *Pseudomonas* infections. Neutralization of virulence factors such as exotoxin A is among promising strategies to control bacterial infections. Moreover, this strategy might affect drug resistance by maintaining the endogenous host-microbiome and creating less selective pressure on the endogenous bacteria [18]. Until now, various antibodies have been developed against different antigens of *Pseudomonas*, but none of them have been approved for clinical use [19].

Theoretically, antibody libraries contain various synthetic and semi-synthetic antibodies, facilitating the isolation of antibodies against any given antigens. At present, scFv phage libraries have an impactful payload in developing monoclonal antibodies without the need for experimental animals [20, 21].

*Pseudomonas* ExoA-DI has been shown to be responsible for the binding of toxin to animal cell receptors and plays a crucial role in the toxicity caused by *Pseudomonas* exotoxin A. The aim of this study was to develop a fully human antibody against domain I of exotoxin A for potential use in neutralizing the toxic effects of *Pseudomonas* exotoxin.

In this study, a novel screening strategy was used for the elimination of nonspecific clones coupled with enrichment of specific clones during biopanning rounds. In this respect, monitoring output phage titers during screening rounds indicated a significant enrichment toward increasing specificity (64 fold) to EoA-DI. During six biopanning rounds, two specific clones with correct VH-VL regions were identified from the fifth round. One of the scFv clones (C9) with high affinity was selected for further studies. In order to generate antibody with high efficiency, the expression and purification of C9 scFv was again performed in *E. coli.* In a 2019 study by Sirijan Santajit et al., they produced human antibodies against subdomain Ia of exotoxin A of *Pseudomona*s using phage display technology [22]. However, in the present study, both subdomains Ia and Ib was used for isolation of human scFv antibody. Although the exact function of domain Ib has not been identified, it has been suggested that this part of exotoxin might be involved in cell recognition [23] or ADP ribosylation [24]. An older study also produced a mouse antibody against exotoxin A, but due to the adverse immune responses related to mouse antibodies for humans, we decided to produce a completely human antibody [25]. S. Nathan [26] used phage display technology for isolation of the antibody against *Burkholderia pseudomallei* by multiple biopanning rounds. They observed high similarity (93%) between different clones, and the main difference between the sequences of clones was related to the CDR3 area. In our study, the highest similarity was 93.9%, and the different area in the sequences was related to CDRs [27].

The scFv clone 9 was purified with high solubility from periplasmic fraction. Based on ELISA results, the resultant anti-exotoxin A scFv specifically recognized both recombinant ExoA-DI and native exotoxin of *P. aeruginosa* with high affinity.

## Conclusion

In this study, we generated and characterized a human anti-exotoxin A scFv with a significant binding potency to exotoxin A of *P. aeruginosa*. This scFv can potentially be considered for developing new therapeutic agents against *P. aeruginosa i*nfections.

## Data Availability

The datasets used and/or analysed during the current study are available from the corresponding author on reasonable request.

## Abbreviations

*scFv*: Single-chain variable fragment
*P*: *Pseudomonas*
*E. coli*: *Escherichia coli*
Ni-NTA: Nickel-NTA
MRC: The Medical Research Council
IPTG: Isopropyl β-D-1-thiogalactopyranoside
LB: Luria-Bertani
SDS-PAGE: Sodium dodecyl sulphate polyacrylamide gel electrophoresis
PBS: *Phosphate-buffered saline*
BSA: Bovine Serum Albumin
OD: Optical density
ELISA: Enzyme linked immunosorbent assay
PCR: *Polymerase chain reaction*
MW: Molecular Wight
kDa: Kilo Dalton
ExoA-DI: Exotoxin A domain I
Exo A: Exotoxin A
PVDF membrane: Polyvinylidene fluoride
anti-M13-HRP: *Horseradish Peroxidase* conjugated *anti*-*M13*
DAB: Diaminobenzidine
